# Potential gains in health-adjusted life expectancy from reducing four main non-communicable diseases among Chinese elderly

**DOI:** 10.1186/s12877-019-1032-3

**Published:** 2019-01-18

**Authors:** Xiaoqian Hu, Xueshan Sun, Yuanyuan Li, Yuxuan Gu, Minzhuo Huang, Jingming Wei, Xuemei Zhen, Shuyan Gu, Hengjin Dong

**Affiliations:** Center for Health Policy Studies, School of Public Health, Zhejiang University School of Medicine, Zijingang Campus, 866 Yuhangtang Rd, Hangzhou, 310058 China

**Keywords:** Health-adjusted life expectancy, Chronic disease, Elderly, Chinese, Burden of disease

## Abstract

**Background:**

To estimate the potential gains in health-adjusted life expectancy (HALE) after hypothetical elimination of four non-communicable diseases (NCDs) among Chinese elderly from 1990 to 2016, including cardiovascular diseases (CVD), cancers, chronic respiratory diseases (CRD) and diabetes mellitus (DM).

**Methods:**

Based on data from Global Burden of Disease 2016, we generated life table by gender using Sullivan method to calculate HALE. Disease-deleted method was used to calculate cause-elimination HALE, after hypothetical elimination of specific diseases.

**Results:**

From 1990 to 2016, HALE increased for all age groups. After hypothetic eliminating the four main NCDs, potential gain in HALE by CVD, DM and cancers increased while by CRD decreased from 1990 to 2016 for both genders. Among four main NCDs, potential gain in HALE after eliminating CVD was largest and increased most for both genders. Although elimination of DM led to the smallest gain in HALE, the increasing speed of gain in HALE by DM was faster than that by CVD and cancers from 1990 to 2016.

**Conclusions:**

This study highlights the potential gains in HALE of NCDs among Chinese elderly from 1990 to 2016. HALE of Chinese elderly could further increase from the reduction of NCDs. Control measures and targeted prevention should be carried out.

**Electronic supplementary material:**

The online version of this article (10.1186/s12877-019-1032-3) contains supplementary material, which is available to authorized users.

## Background

Non-communicable diseases (NCDs) are part of the major health and development challenges in the worldwide. As the leading cause of death globally, NCDs were responsible for 39.5 million (70%) of 56.4 million deaths in 2015 [1]. In China, NCDs are also intractable health problems especially among elders who were suffered heavy disease burden from NCDs [2]. According to results from Global Burden of Disease Study 2016, about 88.5% of all deaths in China were attributable to NCDs, what’s more, 94.0% of the elderly aged over 70 years died from NCDs [3]. The Fifth National Health Services Survey of China reported that the prevalence of NCDs was 33.1% in 2013, compared to 20.7% in 1993. And older people experiences large proportion of burden from NCDs, due to the vulnerable to diseases [4]. With age increasing, morbidity rate rose rapidly. 78.0% people aged over 65 years suffered from NCDs [5]. Meanwhile, due to improvement in medical treatment and better availability of medical care, mortality declined [6] and life expectancy (LE) increased from 43.39 years in 1950 to 75.43 years in 2015 [7]. The improvement in longevity, increasing scale of elderly, together with rising prevalence of NCDs, led to considerable disease burden and concern about the quality of life among elderly people.

The WHO 2008–13 action plan for NCDs focuses on four key diseases, cardiovascular diseases (CVD), cancers, chronic respiratory diseases (CRD) and diabetes mellitus (DM), which cause about 60% of all deaths globally every year [8]. China faces the same even more serious situation. In 2014, CVD, CRD, DM and cancers account for 81% of total deaths, while other NCDs only account for 6% in total [9]. CVD, as the leading cause of death in China [3], are major and growing contributors to mortality and disability. In 2016, mortality attributable to CVD is 309.33 per 100,000 which account for 45.5% of all deaths for rural residents. The corresponding prevalence and proportion for urban residents is 265.11 per 100,000 and 43.2%, respectively [10]. With increasing incidence and mortality, cancer is also a major public health challenge in China. Nearly 22% of global new cancer cases and 27% of global cancer deaths occur in China [11]. In 2016, mortality attributable to cancers is 155.83 per 100,000 which account for 22.9% of all deaths for rural residents. The corresponding prevalence and proportion for urban residents is 160.07 per 100,000 and 26.1%, respectively [10]. CRD, of which chronic obstructive pulmonary disease (COPD) is a major component, also represents a substantial health-care burden in China. In 2016, mortality attributable to CRD is 81.72 per 100,000 which accounts for 12.0% of all deaths for rural residents. The corresponding prevalence and proportion for urban residents is 69.03 per 100,000 and 11.2%, respectively [10]. Compared to other three key diseases, although DM has a relatively smaller impact on mortality, living with it decreases quality of life over the long term [12]. With over 114 million adults suffering from DM (mainly type 2), China now has the largest diabetes epidemic over the world [13, 14]. In 2013, nearly 10.9% of Chinese adults had diabetes [13], exceeding the average prevalence of diabetes worldwide, however the estimated Chinese prevalence of diabetes in 1980 was only 0.67% [15]. DM is the second common disease among Chinese elderly, over 11% urban elderly and 4.5% rural elderly lived with DM in 2012 [5].

Health-adjusted life expectancy (HALE) is a comprehensive indicator, which can reflect the length of and the quality of life simultaneously [16]. Meanwhile it’s a useful indicator to evaluate disease burden of diseases, which were recommended by World Health Organization [17]. Potential gains in HALE resulting from the elimination of deaths and disability from a specific disease could combine the impact of mortality and morbidity, which are particularly useful for estimating the impact of the disease and setting priorities for health planning [18]. It’s important and necessary to monitor the impact of NCDs on HALE to show their influence on life span and life quality simultaneously, especially among elderly with higher prevalence of NCDs and heavier diseases burden. Potential gain in different forms of health expectancy after elimination of diseases was used to show comprehensive pictures of disease burden and had been investigated in a large number of countries [19–22]. However, very few studies assessed the impact of NCDs on HALE in China, especially among older people. Researchers [23, 24] estimated the impact on LE caused by deaths from various diseases, but they neither showed the impact on HALE nor aimed at elderly. Thus, this research aims to estimate the potential gains in HALE after hypothetical elimination of four main NCDs among Chinese elderly from 1990 to 2016, including CVD, cancers, CRD and DM.

## Methods

### Data sources

Data of age-specific population for males and females was from Statistical Yearbook of China 1991 and 2017 [10, 25]. All-cause and specific-cause mortality of people was from Global Burden of Disease Study 2016 (GBD 2016) [3]. Detailed information can be found in previous publications [26]. In brief, with consistent and comparable method, GBD 2016 analyzed the burden of 333 diseases, injuries of 195 countries by age, sex and region from 1990 to 2016 [27]. The data sources of estimation of China in GBD 2016 are extensive, including Disease Surveillance Points, Maternal and Child Surveillance System, Chinese Center for Disease Control and Prevention, Cause of Death Reporting System, National Health Services Survey, and China Health and Retirement Longitudinal Study, and so on since 1990 [28]. In addition, GBD 2016 reviewed the published literature on the morbidity and the prevalence of all kinds of diseases in China. Bayesian Meta regression, time-dependent Gaussian process regression and other methods were used to estimate the death rate by year, province, sex and age in GBD 2016.

We used Years Lived with Disability (YLD) rates to show the prevalence of disability by sex and age group. Data of all-cause and specific-cause YLD was from GBD 2016 too. In GBD 2016, YLD was calculated as the frequency (prevalence) times the disability weight of the associated sequelae (severity) times the duration of symptoms [29].

The definition of CVD, cancers, CRD and DM in this study was from the GBD cause list [3], which was the essential organizing framework for the analysis of causes of death, as well as disease incidence and prevalence and years lived with disability [27, 30, 31]. This cause list was organized hierarchically into four-level, the set of causes was mutually exclusive and collectively exhaustive at each cause hierarchy [30]. At the first level of the GBD cause list, there were three kinds of causes, which were communicable, maternal, neonatal, and nutritional diseases; NCDs; and injuries. At the second level, these three causes were divided into 21 cause groups, such as cancers (neoplasms), CVD and CRD in our study. The third and fourth level of the cause list provided more disaggregated causes [31]. In this study, CVD was a broad cause group at Level 2, included 10 causes such as rheumatic heart disease and rheumatic heart disease at Level 3 and 5 causes at Level 4. Cancers were at Level 2 and involved 29 causes such as liver cancer and stomach cancer at Level 3 and 11 causes at Level 4. CRD were at Level 2, included 5 causes such as COPD and asthma at Level 3 and 4 causes at Level 4. DM was a single cause at Level 3. The detailed GBD cause list was available in additional file [3] [see Additional file 1].

### Statistical methods

The basic method employed in this research was the construction of life tables using the Chiang method [32] and Sullivan method [33]. Abridged period life tables started from age 60 years. There were six age groups with an open-ended group thereafter (85+).

HALE refers to the number of years that a person at a given age can expect to live in good health, taking into account mortality and disability. Using methods developed by Sullivan [34], HALE was calculated by age group within abridged period life table [27].

$$ {HALE}_i=\frac{1}{l_i}\left[\sum \limits_{i=x}^w{L}_i\times \left(1-{YLD}_{r_i}\right)\right] $$

where *l*_*i*_ is the number of persons surviving to exact age *x*_*i*_, *L*_*i*_ is the total number of person-years lived in the age interval (*x*_*i*_, *x*_*i* + 1_), and ${YLD}_{r_i}$ is all-cause disability prevalence in the age interval (*x*_*i*_, *x*_*i* + 1_), ω is the last age group in the life table .

After eliminating a disease, the overall mortality rates and prevalence of disability would reduce, leading to an increase in HALE [18]. The key to estimate the potential gain in HALE from specific disease is to calculate the cause-deleted death probability and cause-deleted disability prevalence [18]. As described in previous studies [18, 35], the effect on HALE of eliminating a disease in this study is calculated assuming independence among causes of death and disability.

The cause-deleted probabilities of death in each age interval were evaluated with cause-elimination life tables [32]. The cause-deleted probability of death (*q*′_*i*_) in the age interval (*x*_*i*_, *x*_*i* + 1_) was calculated using the following formula [36]:

$$ q{\prime}_i=1-{\left(1-{q}_i\right)}^{\left({D}_i-{D}_{ik}\right)/{D}_i} $$

where *q*_*i*_ is the probability of death by all cause in the age interval (*x*_*i*_, *x*_*i* + 1_), *D*_*i*_ is the total number of all deaths in the age interval (*x*_*i*_, *x*_*i* + 1_), and *D*_*ik*_ is the number of deaths attributed to cause *k* in the age interval (*x*_*i*_, *x*_*i* + 1_).

The cause-deleted disability prevalence was calculated from subtracting the specific-cause disability prevalence from all-cause disability prevalence [18]. Then the cause-elimination HALE (CEHALE) was calculated using the cause-deleted disability prevalence in the cause-elimination life tables by Sullivan’s method [18, 33].

$$ {\mathrm{CEHALE}}_i=\frac{1}{l_{ki}}\left[\sum \limits_{i=x}^w{L}_{ki}\times \left\{1-\left({YLD}_{r_i}-{YLD}_{r_{ik}}\right)\right\}\right] $$

where *l*_*ki*_ is the number of persons surviving to exact age *x*_*i*_ after eliminating number of deaths attributed to cause *k*, *L*_*ki*_ is the number of person-years lived in the age interval (*x*_*i*_, *x*_*i* + 1_) after eliminating number of person-years attributed to cause *k*, ${YLD}_{r_i}$and ${YLD}_{r_{ik}}$ are all-cause disability prevalence and specific-cause disability prevalence in the age interval (*x*_*i*_, *x*_*i* + 1_), ω is the last age group in the life table. These functions are calculated in the cause-elimination life tables using the cause-deleted probabilities of death (*q*′_*i*_).

The difference between CEHALE and HALE in each age interval was the potential gain after eliminating a specific disease in HALE (*∆*HALE).

$$ \Delta  {\mathrm{HALE}}_i={CEHALE}_i-{HALE}_i $$

## Results

From 1990 to 2016, HALE increased for both genders in every age group. And females’ HALE increased more than males’ during the past 26 years, which led to enlarged gaps in HALE between genders. For 60-year-old males, HALE increased 3.12 years. The corresponding HALE for females increased 4.31 years. For 80-year-old elderly, HALE increased 1.49 years and 2.08 years for males and females, respectively. (Tables 1-2).

**Table 1:** HALE, CEHALE (with 95% confidence intervals) and potential gain in HALE (with increasing proportion^*^) after eliminating four NCDs in 1990

Age group	HALE without elimination	Elimination of							
		CVD		CRD		Cancers		DM	
		CEHALE	∆HALE	CEHALE	∆HALE	CEHALE	∆HALE	CEHALE	∆HALE
Males									
60–64	12.35 (11.54, 13.06)	15.78 (14.84, 16.61)	3.43 (27.77)	14.24 (13.34, 15.03)	1.89 (15.30)	13.86 (12.96, 14.65)	1.51 (12.23)	12.52 (11.17, 13.19)	0.17 (1.38)
65–69	9.52 (8.84, 10.12)	12.69 (11.88, 13.41)	3.17 (33.30)	11.34 (10.57, 12.02)	1.82 (19.12)	10.69 (9.94, 11.36)	1.18 (12.39)	9.66 (9.01, 10.23)	0.14 (1.47)
70–74	7.04 (6.49, 7.53)	9.91 (9.24, 10.52)	2.87 (40.77)	8.76 (8.11, 9.34)	1.72 (24.43)	7.90 (7.29, 8.44)	0.86 (12.22)	7.15 (6.63, 7.61)	0.11 (1.56)
75–79	5.37 (4.92, 5.79)	7.88 (7.31, 8.41)	2.51 (46.74)	6.89 (6.34, 7.39)	1.52 (28.31)	5.93 (5.44, 6.38)	0.56 (10.43)	5.46 (5.03, 5.85)	0.08 (1.49)
80–84	3.97 (3.60, 4.30)	6.14 (5.65, 6.58)	2.17 (54.66)	5.28 (4.82, 5.70)	1.32 (33.25)	4.30 (3.91, 4.66)	0.34 (8.56)	4.03 (3.67, 4.35)	0.06 (1.51)
85+	2.57 (2.31, 2.80)	4.58 (4.19, 4.92)	2.01 (78.21)	3.77 (3.41, 4.09)	1.20 (46.69)	2.83 (2.54, 3.08)	0.26 (10.12)	2.61 (2.36, 2.84)	0.04 (1.56)
Females									
60–64	14.38 (13.33, 15.32)	18.72 (17.47, 19.83)	4.34 (30.18)	16.35 (15.18, 17.40)	1.98 (13.77)	15.47 (14.34, 16.48)	1.09 (7.58)	14.62 (13.63, 15.51)	0.24 (1.67)
65–69	11.29 (10.40, 12.09)	15.36 (14.26, 16.33)	4.07 (36.05)	13.20 (12.18, 14.11)	1.91 (16.92)	12.15 (11.19, 13.01)	0.86 (7.62)	11.49 (10.64, 12.25)	0.20 (1.77)
70–74	8.55 (7.82, 9.20)	12.29 (11.36, 13.11)	3.74 (43.74)	10.35 (9.49, 11.12)	1.81 (21.17)	9.19 (8.41, 9.88)	0.64 (7.49)	8.70 (8.01, 9.33)	0.16 (1.87)
75–79	6.44 (5.85, 6.98)	9.81 (9.02, 10.51)	3.37 (52.33)	8.06 (7.35, 8.70)	1.62 (25.16)	6.87 (6.24, 7.44)	0.43 (6.68)	6.56 (6.00, 7.07)	0.12 (1.86)
80–84	4.73 (4.25, 5.16)	7.76 (7.08, 8.36)	3.03 (64.06)	6.15 (5.55, 6.69)	1.42 (30.02)	5.00 (4.49, 5.45)	0.27 (5.71)	4.81 (4.35, 5.23)	0.08 (1.69)
85+	3.25 (2.89, 3.57)	6.07 (5.51, 6.57)	2.82 (86.77)	4.56 (4.08, 4.98)	1.31 (40.31)	3.45 (3.07, 3.79)	0.20 (6.15)	3.31 (2.96, 3.62)	0.06 (1.85)

*Note:* ^*^ *This increasing proportion means the ratio of ∆HALE to HALE without elimination in the corresponding age interval*

*HALE health-adjusted life expectancy, CEHALE cause-elimination health-adjusted life expectancy, ∆HALE potential gain in HALE after eliminating a disease, NCDs non-communicable diseases, CVD cardiovascular diseases, CRD chronic respiratory diseases, DM diabetes mellitus*

**Table 2:** HALE, CEHALE (with 95% confidence intervals) and potential gain in HALE (with increasing proportion^*^) after eliminating four NCDs in 2016

Age group	HALE without elimination	Elimination of							
		CVD		CRD		Cancers		DM	
		CEHALE	∆HALE	CEHALE	∆HALE	CEHALE	∆HALE	CEHALE	∆HALE
Males									
60–64	15.47 (14.36, 16.43)	21.51 (19.99, 22.50)	6.04 (39.04)	16.72 (15.48, 17.62)	1.26 (8.14)	17.65 (16.29, 18.64)	2.19 (14.16)	15.82 (14.68, 16.65)	0.35 (2.26)
65–69	12.35 (11.39, 13.21)	18.21 (16.81, 19.08)	5.86 (47.45)	13.61 (12.48, 14.38)	1.26 (10.20)	14.19 (12.97, 15.03)	1.84 (14.90)	12.68 (11.66, 13.39)	0.33 (2.67)
70–74	9.65 (8.83, 10.38)	15.28 (14.00, 16.02)	5.62 (58.24)	10.88 (9.88, 11.53)	1.22 (12.64)	11.10 (10.05, 11.79)	1.45 (15.03)	9.95 (9.05, 10.54)	0.29 (3.01)
75–79	7.33 (6.65,7.94)	12.67 (11.52, 13.30)	5.34 (72.85)	8.48 (7.63, 9.01)	1.15 (15.69)	8.42 (7.54, 8.97)	1.09 (14.87)	7.59 (6.83, 8.06)	0.26 (3.55)
80–84	5.46 (4.90, 5.95)	10.52 (9.49, 11.06)	5.06 (92.67)	6.52 (5.79, 6.95)	1.06 (19.41)	6.25 (5.52, 6.68)	0.79 (14.47)	5.67 (5.04, 6.05)	0.21 (3.85)
85+	4.02 (3.57, 4.40)	8.92 (7.99, 9.39)	4.90 (121.89)	5.00 (4.40, 5.34)	0.99 (24.63)	4.64 (4.06, 4.97)	0.62 (15.42)	4.19 (3.68, 4.48)	0.17 (4.23)
Females									
60–64	18.69 (17.21, 20.02)	26.07 (24.28, 27.71)	7.39 (39.54)	19.84 (18.32, 21.22)	1.15 (6.15)	19.99 (18.41, 21.42)	1.31 (7.01)	19.06 (17.66, 20.33)	0.37 (1.98)
65–69	15.20 (13.90, 16.36)	22.41 (20.79, 23.90)	7.22 (47.50)	16.32 (14.98, 17.54)	1.13 (7.43)	16.29 (14.90, 17.53)	1.09 (7.17)	15.52 (14.29, 16.63)	0.32 (2.11)
70–74	12.02 (10.92, 13.01)	19.00 (17.56, 20.33)	6.99 (58.15)	13.10 (11.95, 14.15)	1.08 (8.99)	12.88 (11.72, 13.95)	0.87 (7.24)	12.28 (11.24, 13.24)	0.27 (2.25)
75–79	9.19 (8.30, 10.02)	15.89 (14.64, 17.06)	6.70 (72.91)	10.21 (9.26, 11.09)	1.02 (11.10)	9.85 (8.91, 10.73)	0.66 (7.18)	9.40 (8.55, 10.19)	0.21 (2.29)
80–84	6.81 (6.10, 7.48)	13.23 (12.14, 14.27)	6.42 (94.27)	7.75 (6.98, 8.48)	0.94 (13.80)	7.29 (6.54, 8.00)	0.48 (7.05)	6.97 (6.29, 7.61)	0.16 (2.35)
85+	4.89 (4.36, 5.40)	11.11 (10.19, 12.01)	6.22 (127.20)	5.75 (5.16, 6.33)	0.87 (17.79)	5.27 (4.71, 5.81)	0.38 (7.77)	5.01 (4.50, 5.50)	0.12 (2.45)

*Note:* ^*^ *This increasing proportion means the ratio of ∆HALE to HALE without elimination in the corresponding age interval*

*HALE health-adjusted life expectancy, CEHALE cause-elimination health-adjusted life expectancy, ∆HALE potential gain in HALE after eliminating a disease, NCDs non-communicable diseases, CVD cardiovascular diseases, CRD chronic respiratory diseases, DM diabetes mellitus*

In 1990, females had longer HALE than males in corresponding age groups. Elimination of CVD led to the greatest gains in HALE between both genders, followed by CRD and cancers. The gains in HALE from DM were smallest. For 60-years-old elderly, the potential gains from CVD were nearly twice as large as that from CRD, and nearly 20 times as large as that from DM. Females had greater gains in HALE from CVD, CRD and DM than males in corresponding age groups, while gains in HALE for males from cancers were larger. (Table 1).

In 2016, females had longer HALE than males in corresponding age groups. The potential gains in HALE from CVD still were the largest among four NCDs for both genders. The gains in HALE from cancers were the second large, which exceeded that from CRD. DM still caused smallest gains in HALE. Females had greater gains in HALE from CVD than males in corresponding age groups, while eliminating of CRD and cancers led to larger gains in HALE for males. Males had more gains in HALE from DM than females on almost age groups. (Table 2).

From 1990 to 2016, potential gains in HALE after eliminating CVD, cancers and DM increased for both males and females, while potential gains in HALE after eliminating CRD decreased which may because CRD was far less prevalent in 2016 than in 1990, coinciding with declining smoking rates. The gains in HALE from CVD nearly doubled from 1990 to 2016 among the Chinese elderly. Although gains from DM were smallest, they increased fastest among four NCDs. For males, the gains in HALE from cancers and DM increased more than females. By contrast, the gains from CVD for females increased more than males. And the gains from CRD for females decreased more than males indicating a lower disease burden from CRD on females than it on males. The possible reason may be the lower smoking rates among females. These different changing extents led to enlarged gaps between genders. (Tables 1-2).

## Discussion

In this study, we used data from GBD 2016 to estimate the potential gains in HALE by four main NCDs from 1990 to 2016 among the Chinese elderly. The results showed that from 1990 to 2016, HALE increased for all age groups. After hypothetic eliminating the four main NCDs, potential gains in HALE by CVD, DM and cancers increased while by CRD decreased for both genders. Among four main NCDs, potential gains in HALE after eliminating CVD were largest and increased most during the past 26 years for both genders. Although elimination of DM led to the smallest gain in HALE, the increasing speed of gain in HALE by DM was faster than that by CVD and cancers from 1990 to 2016.

CVD caused largest potential gain in HALE for males and females in 1990 and 2016, which was the major barrier to increase both the length of and the quality of life among elderly deserving more effort to control it. A Germany study pointed that the largest contribution to increases in life expectancy came from the decline of CVD mortality [37]. There were similar findings from the United States and other European countries [38, 39]. The reasons for the accelerated mortality declining were the effective management of major risk factors like blood pressure, alcohol and the realization of universal coverage [40]. Thus, through timely prevention and effective treatment of CVD, we could also reduce its incidence, mortality and disability rate to pursue healthier life in China [24].

Although the potential gain in HALE by DM was not as high as CVD, the impact of DM on HALE increased quickly from 1990 to 2016, which may because that DM is not as fatal as CVD [41]. Mostly DM didn’t cause deaths directly, thus there may be underestimation of its effect on HALE which combined mortality and morbidity together [41]. People with DM, suffering from complications [42] and poor glucose control [42] were easier to be disability, which affected the quality of life seriously. Great importance still needs to attach on prevention of DM in case it would cause larger impact on health among Chinese elderly further. Elimination of DM leading to increasing gain in HALE indicated an urgent need to raise concern about DM through intervention and prevention. There was growing evidence that DM and its complications could be prevented through early detection and reduction of key risk factors like physical inactivity, obesity and immoderate diet [12].

Among four NCDs in 2016, the potential gains in HALE from CRD, cancers and DM for males were larger than females, indicating interventions about CRD, cancers and DM could target males as priority population. Previous researches also founded the similar results [12]. And the potential gains in HALE from CVD were larger for females than males, indicating more attention should be paid on the prevention of CVD among females. Meanwhile, for both males and females, the greatest gains in HALE would occur by preventing NCDs in the youngest age groups, and 60–69 for example might be the best group for targeted interventions.

Although the causes of NCDs were complex, through lifestyle transition and timely prevention from the early life, NCDs could be prevented or postponed to older ages [23]. Lots of studies noted that many NCDs shared common risk factors such as high blood pressure, tobacco use, cholesterol and physical inactivity [43]. And these factors are modifiable by early education and long-term prevention at the individual and population level [44, 45]. Finland carried out comprehensive community-based interventions which were aimed at changing the target risk factors and health behaviors (like serum cholesterol, blood pressure, smoking, and diet) at the population level. As a result, there was a great reduction in death from NCDs [46]. Lots of similar successful cases in other countries [40, 47] could be promoted in China. Previous programs in NCDs prevention and control in China also achieved effective results, which should be strengthened and spread further [45]. ‘Healthy China 2030’, which was the first medium and long term strategic project in the field of health at the national level, emphasized that using a life course concept to control NCDs, lots of actions should be taken during the whole life. Interventions aimed at ensuring a healthy body composition, adequate nutrition, and lifestyle before pregnancy could have powerful influence on reducing the incidence of DM and other NCDs [48]. In childhood, education about healthy lifestyle and diet should be underlined that would benefit people not only temporarily but also throughout life. In adulthood, people are encouraged to keep healthy lifestyles and regular medical checkup to prevent themselves from NCDs. When at old age, timely diagnosis and treatment are necessary [44]. Thus, there is an urgent need for strategies and policies that prevent NCDs by reducing those major risk factors beginning from the early life, together with implementing a universal, strong and equitable primary health care system [49].

There are some limitations in this research. First, the ‘complete’ elimination of the deaths and disability due to these NCDs was hypothetical and not realistic, due to it was impossible to eliminate all upstream associated risk factors from a public health perspective. While this artificial hypothesis was still very useful in identifying the maximum potential gains in HALE by targeted interventions. Second, this study assumed that these four NCDs were mutually exclusive or independent. However, this assumption may not be realistic, because these NCDs sharing many common risk factors. Limited to available data, we could not calculate HALE of elderly with and without specific NCDs. The difference between those elderly groups with and without diseases would reveal the impact of specific diseases on health better and more realistic. Hence it is necessary to establish surveillance systems of risk factors and NCDs around China. Third, due to the higher prevalence of and heavier diseases burden from NCDs among Chinese elders, this study aimed to explore the potential gains in HALE from old age. Further studies could extend the research range to the impact on HALE at birth. Fourth, due to the limited data, this research could not estimate different potential gains in HALE in different regions, such as urban area and rural area. What’s more, this research didn’t subdivide NCDs into several specific diseases. Further studies may add these aspects.

## Conclusions

In conclusion, HALE of Chinese elderly could further increase from the reduction of NCDs. Future health care planning and prevention strategies for NCDs should be taken for the Chinese to own healthy lives.

## Additional file

Additional file 1: GBD cause list. This additional file 1 included the details of the GBD cause list. (XLSX 25 kb)

## Abbreviations

CEHALE: cause-elimination health-adjusted life expectancy
COPD: chronic obstructive pulmonary disease
CRD: chronic respiratory diseases
CVD: cardiovascular diseases
DM: diabetes mellitus
GBD: Global Burden of Disease Study
HALE: health-adjusted life expectancy
LE: life expectancy
NCD: non-communicable diseases
YLD: Years Lived with Disability
